# Assessment of open data kit mobile technology adoption to enhance reporting of supportive supervision conducted for oral poliovirus vaccine supplementary immunization activities in Nigeria, March 2017-February 2020

**DOI:** 10.11604/pamj.supp.2023.45.2.38140

**Published:** 2023-07-14

**Authors:** Philip Bammeke, Tesfaye Erbeto, Aron Aregay, Zabihullah Kamran, Usman Said Adamu, Eunice Damisa, Nnamdi Usifoh, Peter Nsubuga, Ndadilnasiya Waziri, Omotayo Bolu, Edward Dagoe, Faisal Shuaib

**Affiliations:** 1 Centers for Disease Control and Prevention, Atlanta, United States,; 2 World Health Organization, Abuja, Nigeria,; 3 World Health Organization, Kyiv, Ukraine,; 4 United Nations Children´s Fund, Copenhagen, Denmark,; 5 National Primary Health Care Development Agency, Abuja, Nigeria,; 6 Global Public Health Solutions, Atlanta, United States,; 7 African Field Epidemiology Network, Abuja, Nigeria

**Keywords:** Open Data Kit, supportive supervision, polio eradication, mobile technology, real-time data, supplementary immunization activities, vaccination campaign, oral poliovirus vaccine, digital health

## Abstract

**Introduction:**

in Nigeria, supportive supervision of Supplementary Immunization Activities (SIA) is a quality improvement strategy for providing support to vaccination teams administering the poliovirus vaccines to children under 5 years of age. Supervision activities were initially reported in paper forms. This had significant limitations, which led to Open Data Kit (ODK) technology being adopted in March 2017. A review was conducted to assess the impact of ODK for supervision reporting in place of paper forms.

**Methods:**

issues with paper-based reporting and the benefits of ODK were recounted. We determined the average utilization of ODK per polio SIA rounds and assessed the supervision coverage over time based on the proportion of local government areas with ODK geolocation data per round.

**Results:**

a total of 17 problematic issues were identified with paper-based reporting, and ODK addressed all the issues. Open Data Kit-based supervision reports increased from 3,125 in March 2017 to 51,060 in February 2020. Average ODK submissions for national rounds increased from 84 in March 2017 to 459 in February 2020 and for sub-national rounds increased from 533 in July 2017 to 1,596 in October 2019. Supportive supervision coverage improved from 42.5% in March 2017 to 97% in February 2020.

**Conclusion:**

the use of digital technologies in public health has comparative advantages over paper forms, and the adoption of ODK for supervision reporting during polio SIAs in Nigeria experienced the advantages. The visibility and coverage of supportive supervision improved, consequentially contributing to the improved quality of polio SIAs.

## Introduction

Eradication of the poliomyelitis virus (polio) has been a global public health priority since 1988 [1]. The resolution in 1988 by the Global Polio Eradication Initiative (GPEI) to eradicate polio globally led to the adaptation of appropriate strategies developed in the World Health Organization (WHO) Region of the Americas to achieve this goal. This included implementing mass vaccination campaigns in the form of national immunization days, sub-national immunization days, and mop-up activities to vaccinate all targeted children within a short time [2]. These campaigns are critical in polio-endemic countries with weak or low routine immunization (RI) coverage [3,4]. Polio mass vaccination campaigns, which are Supplemental Immunization Activities (SIA) provide opportunities to boost polio vaccine exposure and improve suboptimal immunity achieved through RI. Oral poliovirus vaccine (OPV) SIAs also provide the avenue for additional polio vaccine administration to children who have completed or partially completed their RI OPV schedule [5]. During SIAs, vaccination teams move from house-to-house and station at strategic public places to administer OPV doses to all children under five years of age, regardless of previous vaccination status [6,7]. Conducting quality polio SIAs is vital to boost children´s immunity in all areas and interrupt wild poliovirus transmission and maintain polio-free status [5,8]. To reach the highest possible coverage during SIAs, detailed planning, meticulous execution, careful supervision, and performance monitoring are critical activities [2].

Nigeria was the last country in the WHO African Region (AFR) with wild poliovirus (WPV) transmission, reported in 2016. With the successful interruption of WPV transmission in Nigeria, WHO-AFR was certified WPV-free in August 2020, with only Afghanistan and Pakistan remaining WPV-endemic [9,10]. Several strategies contributed to the significant milestone achieved in 2020 by the Nigerian Polio Eradication Initiative (PEI) program. Noteworthy was the establishment of the National Polio Emergency Operations Center (NEOC) in 2012 by the Nigerian Government with the support of partners to coordinate polio eradication activities [11,12]. The NEOC implemented innovative strategies over time, including coordinated deployment of supervisors from the Government and partners to provide supportive supervision and monitoring during polio campaigns.

Before March 2017, paper forms were used to report supervision conducted and reports sent to NEOC after a polio campaign ends. This made monitoring and documentation of supervision visits conducted during campaigns challenging. The paper-based approach limited the use of supervision reports in real-time for data analysis and effective feedback [13]. Open Data Kit (ODK) mobile technology was adopted in March 2017 for supportive supervision reporting in Nigeria to address the limitations of paper forms and consequentially strengthen supervision quality during polio SIAs. Open Data Kit is a free and open-source application on Android for mobile data collection, and an internet connection is not required to enter or capture data [14]. The adoption of ODK was part of the strategies deployed to improve the quality of the polio SIAs with the re-emergence of WPV type 1 in 2016, after going two years without detecting a case in Nigeria [15]. Therefore, this study was done to review the impact of adopting ODK for supportive supervision reporting during polio SIAs in Nigeria from March 2017 and ascertain that the benefits experienced with ODK to strengthen supervision data reporting and management, monitoring of supervision visits, and use of data for action was challenging with paper forms.

## Methods

**Study design:** a comparative cross-sectional study designed to highlight the experience of introducing ODK in place of paper forms for supervision reporting during polio SIAs in Nigeria. To assess the impact of ODK´s adoption, this study was designed to quantify the trend of reporting rate and supervision coverage. The lower year limit of the analysis period was March 2017 when ODK was introduced, while the upper limit was February 2020 when the last polio SIA was conducted before the COVID-19 pandemic lockdown in Nigeria in April 2020 and WPV-free certification of WHO-AFR in August 2020. Only the polio SIAs conducted with the administration of bivalent Oral Polio Vaccine (bOPV) which contains only type 1 and type 3 Sabin strain polioviruses were included in the study [16].

**Setting:** the entire 36 states and the Federal Capital Territory (FCT) of Nigeria, a West-African country along the Gulf of Guinea. Borno state in the northeast of Nigeria around Lake Chad was sampled to further evaluate the impact of ODK use on supervision coverage, while Gombe state also in the northeast of Nigeria was sampled to highlight spatial data triangulation of ODK data with other sources for in-depth analysis such as the pattern of supervision visits during polio SIAs.

**Participants:** supervisors deployed jointly as management support teams from the national level by the government and the GPEI partners for each polio SIA campaign round [17]. Polio human assets from the government and polio partners at the state and Local Government Area (LGA) levels were also deployed as supervisors. The supervisors were often polio program specialists or public health officers that provided technical support and guidance to vaccination teams in the delivery of two doses of OPV to children under five years of age in each campaign. We the members of the Data Working Group (DWG) at the NEOC during 2016-2020 managed the deployment of ODK for reporting of supervision visits conducted during polio SIAs in Nigeria and are the lead researchers of this study. We recounted the benefits experienced with ODK in place of paper forms.

**Variables:** the variables used to assess paper and ODK reporting tools across eight thematic areas were data entry, data submission, data acquisition and processing, data analysis and use, supervision coverage, data triangulation, data management, and paper management related cost. The variables to enumerate the reporting rate with ODK were the month and year of national and sub-national SIAs, the number of states and LGAs involved, the total number of submissions to the ODK server per round, and the average/standardized number of ODK-based submission reports per round. The variables to measure supervision coverage was the proportion of LGAs reached out of the total participating LGAs per round, based on at least one ODK-based submission. For supervision coverage impact in Borno state, the variables were supervision points as of day two during March 2017 and March 2018 SIAs, and LGAs that could not implement due to insecurity were highlighted. For Gombe state´s specific spatial analysis, the variables were type of settlements which were hamlets, small settlements, and built-up areas.

**Data sources:** supervisors deployed for each round of polio SIAs were required to report supervision visits conducted daily using ODK. Each round was for four days, and each supervisor was expected to supervise an average of three teams per day. Supervisors captured data on supervision conducted for each team visited and submitted digital reports daily to the ODK server across the four days. The DWG members described based on direct observation the issues with paper forms and benefits experienced with the ODK to make the qualitative comparative analysis. The submitted ODK-based supervision data for each SIA round was sourced from the dedicated data repository in a secured online server managed by the DWG. The NEOC provided the data on each polio SIA round by month, and the number of states and Local Government Areas (LGAs) that participated in each round. The data on the type of settlements visited in Gombe for the spatial data triangulation was sourced from a system called Vaccination Tracking System (VTS) which is used to monitor planned settlements visited by vaccination teams to administer OPV doses to eligible children during polio SIAs in Nigeria [18].

**Open Data Kit-based form design and management:** the paper forms were converted into digital format for use on ODK-enabled Android mobile devices, applicable in any settlement of the 774 LGAs across the 36 states and the Federal Capital Territory (FCT) of Nigeria. The form automatically captured Global Position System (GPS) coordinates at the beginning and end of each supportive supervision session within a 10-meter accuracy radius. Timestamps were embedded in the form design to automatically calculate and record the actual time and date that a supervisor captured GPS coordinates at the beginning and the end of a supportive supervision session, in the event a supervisor erroneously or intentionally manually entered another time or date. A technical user guide was developed to assist supervisors deployed to the field in using ODK to collect supervision data, with well-illustrated pictorials on the following: 1) installation of ODK App on Android mobile devices from Google Play Store; 2) configuration of server settings on ODK to the secured central server; 3) automatic update of checklist configuration; 4) downloading the required supervision reporting checklist; 5) utilization of ODK checklist to enter data, save and edit captured data; 6) transmitting the captured data on supervision conducted to the central server.

**Bias and control:** the number of states and LGAs across the states that participated in each SIA round varied, so the standardized average submission of ODK-based submissions was used to enumerate the reporting rate per round. The submission rates were reviewed separately for national rounds classified in this study as a round with all states or 80% or more of the states involved, and sub-national rounds classified as a round with selected states involved, often 50% or less.

**Study size:** the efforts to interrupt WPV in Nigeria was a nationwide based on GPEI standards which requires a country to go three years without the detection of a paralytic case caused by WPV infection, as an indication of the confidence that WPV transmission has been interrupted with the assumption that a good polio AFP surveillance system in place [15]. Evaluation of the impact of ODK on SIA supervision reporting and quality over time was nationwide and over at least three years stretch from the last WPV case reported in 2016.

**Data analysis:** for the qualitative comparative analysis, we documented the challenges experienced previously with the paper-based forms across the eight thematic areas and the benefits experienced with ODK that addressed the challenges. For the quantitative analysis, we evaluated the use of ODK by supervisors during March 2017-February 2020 based on the number of submissions for each polio SIA round. To objectively measure the utilization of ODK since the number of participating states varied each SIA round, we estimated the average number of submissions per round by dividing the total submissions over the number of participating states. For the coverage spatial analysis, we calculated the proportion of LGAs reached for supervision based on the number of LGAs with at least an ODK supervisory visit submission (numerator) divided by the number of participating LGAs (denominator) each round. The areas reached for supervision were based on GPS coordinates geolocation data in the ODK reports. The GPS data were mapped using ArcGIS 10.5. Spatial query analysis was conducted to highlight LGAs with at least one GPS coordinate to indicate the LGAs visited for supervision. Borno state was sampled to measure the impact of ODK on supervision coverage within a year interval after adoption in March 2017. Gombe state was used as a sample to perform spatial data triangulation by overlaying supervision geolocation data over the settlement tracks from the VTS to characterize the pattern of settlements visited for supervision.

**Ethical approval:** the NEOC approved the research and the identities of the supervisors that collected the data during polio SIAs were not disclosed or used in this study.

## Results

**Figure 1:**
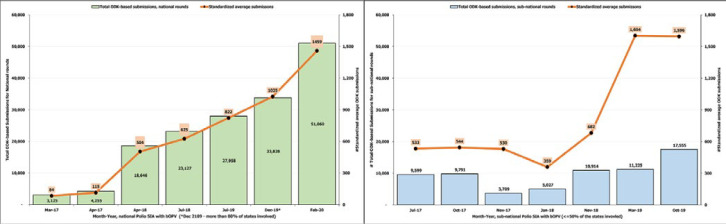
supportive supervision reports submitted with open data kit during polio supplementary immunization activities with bivalent oral polio vaccine from March 2017-February 2020

The qualitative comparative analysis of the two approaches of reporting supervision activities during polio SIAs based on the recounted experience of the DWG highlighted 17 specific issues across the eight thematic areas with the paper forms (Table 1). The benefits recounted with the adoption of ODK from March 2017 indicated all 17 issues were fully or partially addressed. Open Data Kit reporting ensured complete data entry and enabled the submission of supervision reports from any location. Open Data Kit-based reporting markedly enhanced data retrieval for timely data analysis to identify gaps and recommend corrective actions. The GPS data associated with reports submitted to the ODK central server enabled coverage assessment of supportive supervision. The ODK platform improved data management and decreased the processing time to use the data. For the quantitative analysis to enumerate the impact of ODK on the supervision reporting rate over time, there were 14 SIA rounds within the analysis period during March 2017-February 2020 (Table 2). Complete national rounds were 6 and a round in December 2019 was classified also with the national rounds for the study because ≥80% of the states were involved. The other 7 SIA rounds were sub-national with ≤50% of the states involved. The national round in March 2017, when ODK reporting was initiated, had the lowest crude number of submissions (3,125), while the national round in Feb 2020 had the highest number of crude submissions (51,060) (Table 2). The average number of ODK-based report submissions by deployed supervisors for the national SIAs, including the December 2019 round, increased from 84 in March 2017 to 1,459 in February 2020 (Figure 1). The average number of submissions for sub-national SIAs increased from 533 in July 2017 to 1,596 in October 2019 (Figure 1).

**Table 1:** recounted experience by 2016-2020 data working group members of the National Polio EOC on issues with paper forms and benefits of Open Data Kit (ODK) to report supportive supervision conducted during polio Supplementary Immunization Activities (SIA) in Nigeria

	Aspect	Description of issues with paper forms	Benefits of using ODK
1	Data entry	Invalid data - illogical or inconsistent data reporting errors	ODK forms were designed with skip logic and validation rules to improve data quality
		Incomplete data - for the required questions, data sometimes may not be provided	Enforced rules coding to ensure complete data entry
2	Data submission	Loss of captured data during transcription or paper tear	Captured data are stored safely and ready to use
		Prolonged transmission of data - time and distance required to submit reports, especially from remote areas	Transmission of reports to the central server from any location in real-time
3	Data acquisition and processing	Quite daunting to access, retrieve and aggregate reports of supervisors from various locations	ODK aggregate web interface made it faster to retrieve submitted reports in real-time
		Prolonged data transcription process to manually transcribe and accumulate high volume of paper reports	High volume of digital data records that were quickly ready for use, no transcription
		Legibility issues - difficult to read some handwritings	No legibility issues with ODK
4	Data analysis and use	Delayed data availability limited daily data analysis during the polio SIAs	Faster data availability enabled daily data analysis to identify gaps during the polio SIAs
		Poor feedback mechanism including limited sharing of situation reports during the polio SIAs	Situational feedback reports rapidly generated daily during the polio SIAs to guide the use of data for action
5	Supervision coverage	Unable to verify the location of supervision conducted	Geo-coordinates data captured with ODK to map supervision locations
		Difficult to monitor if supervision was widespread or clustered to some areas	Mapping of geocoordinates reveal coverage/pattern of supervision visits
6	Data analytics and triangulation	Unable to integrate supervision data with other data sources for data triangulation and in-depth analysis	ODK geocoordinates data were triangulated with other data sources to make in-depth analysis
		Limited the use of dynamic or intuitive data visuals to communicate situations during the polio SIAs in near real-time	Enabled the rapid use of visuals to aid communication of situations in multiple areas in near real-time
7	Data management	Challenging data archival process and cumbersome to store paper reports that required physical storage	Digital records were stored in a secured online server
		Difficult to make edits and modify paper forms	Able to automatically update digital forms on devices after a modification
8	Paper management related cost	Printing of paper forms has cost implications over time	No printing, the only cost is the Android mobile device
		Inadequate hard-copy data tools - a supervisor may exhaust paper forms and will not capture more required data	ODK blank forms can be reused newly countless times

**Table 2:** Open Data Kit mobile technology reports submitted for supportive supervision conducted during Polio Supplementary Immunization Activities with only bi-valent oral polio vaccine in Nigeria, March 2017-February 2020

	SIA round (month-year)	SIA Scope	# States	# LGAs involved	# Total report submissions
1	March 2017	NIPDs	36 + FCT	772	3,125
2	April 2017	NIPDs	36 + FCT	772	4,255
3	July 2017	SIPDs	17 + FCT	380	9,599
4	October 2017	SIPDs	17 + FCT	380	9,791
5	November 2017	SIPDs	7	133	3,709
6	January 2018	SIPDs	13 + FCT	294	5,027
7	April 2018	NIPDs	36 + FCT	772	18,646
8	July 2018*	NIPDs	36 + FCT	771	23,127
9	November 2018	SIPDs	15 + FCT	287	10,914
10	March 2019	SIPDs	7	142	11,225
11	July 2019	NIPDs	33 + FCT	609	27,958
12	October 19	SIPDs	11	271	17,555
13	December 2019	SIPDs	32 + FCT	489	33,828
14	February 2020	NIPDs	34 + FCT	724	51,060

* 3 states, Adamawa, Borno, and Yobe, conducted the round in March 2018 to synchronize with neighboring Lake Chad countries FCT – Federal Capital Territory NIPDs – National Immunization Plus Days SIPDs – Sub-national Immunization Plus Days (selected states)

**Figure 2:**
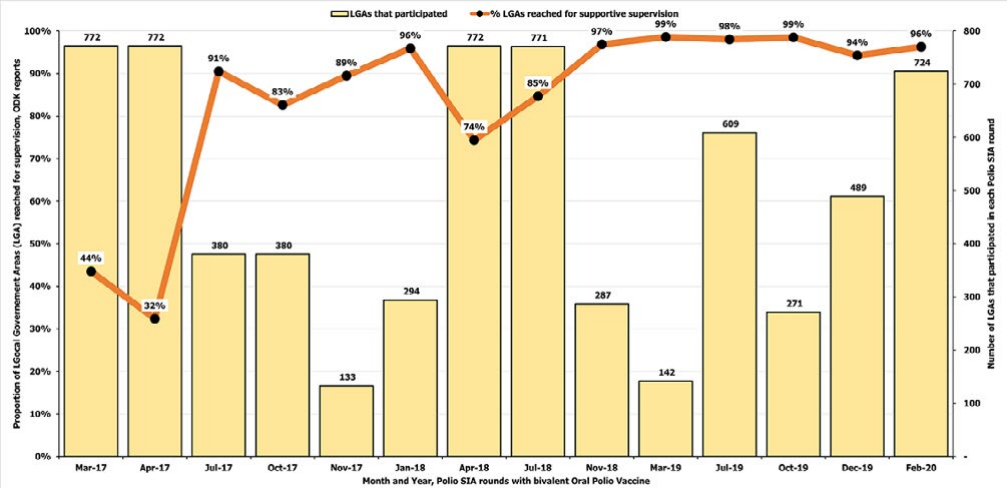
proportion of local government areas that participated in each round of polio supplementary immunization activities reached for supportive supervision based on open data kit reports, March 2017-February 2020

**Figure 3:**
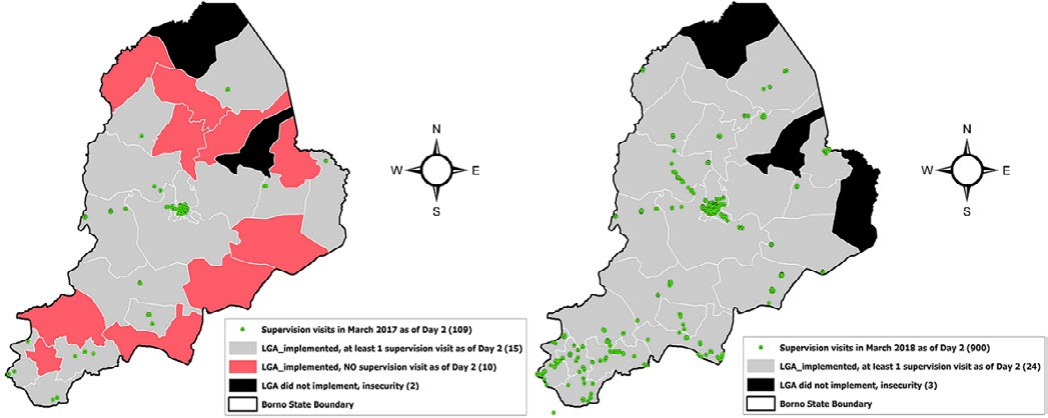
supportive supervision reach in Borno state, Nigeria as of day 2 of March 2017 (left map); polio supplementary immunization activities compared to March 2018 (right map)

**Figure 4:**
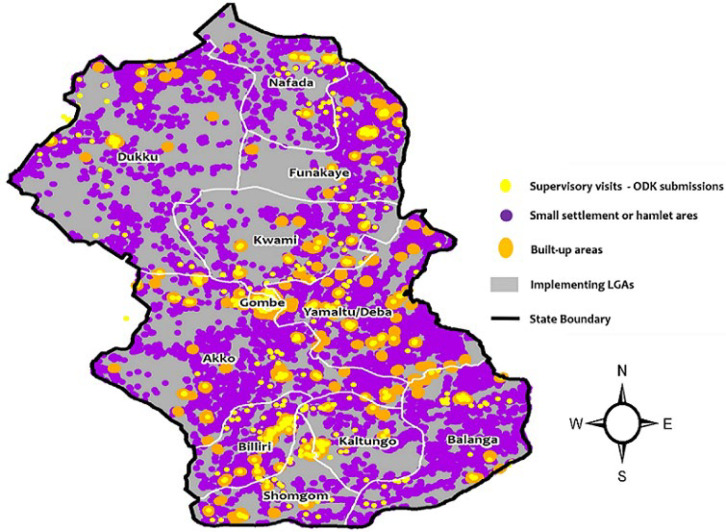
overlay of supervision conducted based on the geolocation in open data kit reports over the type of settlements reached for vaccination based on settlements tracks from the vaccination tracking system in Gombe state, Nigeria during a polio supplementary immunization activities round in 2018

For the spatial analysis, the measurement of the supervision coverage showed that the reported coverage of supportive supervision visits at the LGA level during polio SIAs was low for the first two SIA rounds after the adoption of ODK in March 2017 (42.5% in March 2017; 32.4% in April 2017) (Figure 2). The coverage increased to 90.5% in July 2017. After some fluctuation, the coverage increased to an average of 97.0% for the SIAs from November 2018 to February 2020. In Borno state with insurgency challenges that limited vaccination reach and other polio eradication activities in certain areas of the state, most supervision visits were limited to more accessible central and southern parts of the state in March 2017. During March 2017 polio SIA, 25 out of 27 LGAs could implement and 60% of these 25 LGAs had supervision visit evidence as of day two. For March 2018 polio SIA, 24 out of 27 LGAs could implement and 100% had supervision visit evidence as of day two. ODK-based assessment of the extent of supervision incentivized supervisors to spread and support vaccination where possible and safe towards the northern and eastern parts over time (Figure 3). For the spatial data triangulation during a 2018 round in Gombe, the overlay of the GPS location data for the ODK-based conducted supervision reports over the settlements reached by vaccination teams from the VTS tracks showed the pattern of supervision visits conducted were high skewed to the built-up settlements that often have better living conditions and proximity, to the detriment of small settlements and hamlet areas mostly in rural and remote areas (Figure 4).

## Discussion

Like any public health program, polio eradication needs managerial and technical support, including coordination, performance review, communication, training, adequate resource provision, and supervision [19]. The adoption of ODK as the supportive supervision reporting mechanism for polio SIA campaigns in Nigeria compared to using a paper-based checklist was a valuable migration strategy that yielded immense benefits for operational data on intra-campaign activities. The visibility, accountability, and reach of supportive supervision improved over the years with the use of ODK. The presence of supervisors at the spot of vaccination was a positive indication of the performance of vaccination teams delivering the OPV doses to eligible children during polio SIAs. Open Data Kit adoption in March 2017 was also crucial to the Nigerian polio program because innovative and improvement strategies were needed across the board after the resurgence of WPV cases in 2016.

The timely availability of credible and reliable supportive supervision data and the use of situational data analysis findings to trigger preventive or corrective measures during polio SIAs is critical to the Nigerian PEI program. A paper-based checklist made these difficult to achieve because data reporting and entry are time-consuming, and challenging to prepare, distribute, collate, store, and update. Paper-based checklists are easy to lose or destroy and difficult to replicate, therefore generally not an effective data collection tool on a large scale [20]. The ODK checklist for Nigeria was designed with validation rules to enforce data entry in the appropriate format where required, skip logic based on selected options, and automatic calculation to generate some outputs based on some entered data. In Kenya, ODK was adopted for a large application system that utilized handheld devices to track clinical care home visits in a resource-constrained environment. Data were immediately available electronically, initial reports could be performed, and important trends in data could be easily assessed. The experience in Kenya found ODK to be a cost-effective solution for collecting a large volume of data [21].

With the dynamic digital age, mobile devices are becoming more widely available at economical rates [21,22]. The last few years have witnessed an increase in the use of emerging mobile technologies in healthcare services and programs for better, faster, and more error-free data collection and analysis processes, compared to paper-based modes of information gathering [23]. Open Data Kit is one of such tools that has recently gained more popularity in developing countries because it is an open-sourced platform, easy to deploy and use, and does not need internet connectivity to enter data making it possible to collect data at any time offline with ODK [23,24]. Supervisors deployed for SIAs could afford to operate and enter data without any concern about internet connectivity which may not be stable or available in remote areas. Internet connectivity was only required to install the ODK app from the Google Play store, download the supportive supervision checklist, and transmit finalized form with collected data to the private central server. If any modification to the form is made on the central server, auto-configuration on ODK updates the version of the downloaded checklist once there is an internet connection.

Another advantage of ODK was the acquisition of GPS coordinates included in the data collected by supervisors. The time stamps of GPS data are automatically captured backend and are available in the database for analysis. This is useful in the data cleaning process to eliminate invalid records (e.g. the date and time manually entered by a supervisor cannot be earlier than the GPS capture timestamp at the end of a supervision session). The difference between timestamps of GPS capture at the beginning and the end is used to determine the time spent for supervision so that analysis is not relying on times manually entered by a supervisor. The GPS data from submitted reports on ODK were used to identify the pattern of supervision visits and areas not yet visited for supervision. The GPS data allowed spatial data triangulation with other datasets for in-depth analysis; for example, the overlay of supportive supervision visits on types of settlements visited can be used to determine if supervision is skewed to urban areas which are often more convenient to access with better resources, to the detriment of other types of settlements that are often remote, and most times have more challenges and require more supervision. The GPS data of the digital technology enabled the overlay of supervision locations over VTS tracks of settlements reached by vaccination teams. Data triangulation with other systems provided more in-depth analysis to further assess the credibility of supervisory visits occurring in areas where delivery of vaccines to eligible children took place and to better determine poorly supervised areas or areas where supervisors are clustered.

In Borno state, with insurgency challenges that limited vaccination reach in some regions of the state, ODK enabled the tracking and assessment of supportive supervision conducted in areas accessible to vaccination teams. Initial supervision visits in March 2017 were limited to and clustered around the safer central and southern parts of the state, compared to the northern and eastern parts with more security risk. With the mandatory use of ODK for supervision reporting and broad knowledge of supervision location tracking as the accountability mechanism over time, there was evidence of increased supervision spread to the north and eastern areas of the state that was increasingly accessible to standard vaccination teams. A similar effect of supervision geographic spread also gradually emerged in the other states with increasing participation in polio SIA rounds. This was evident with the substantial increase in the proportion of participating LGAs per each polio SIA round reached for supportive supervision between March 2017 and February 2020. Widespread supportive supervision became one of the indicators of assessing polio SIAs among other indicators. The overall quality of a polio SIA is however assessed by Lot Quality Assurance Sampling (LQAS), a physical check of vaccinated children from sampled lots by independent monitors post-campaign [25,26].

**Limitations:** a limitation of the study was that we did not directly assess the association between supportive supervision and the quality of each SIA as determined by LQAS and any association would have been confounded by other SIA strategies. Another limitation was the number of supervisors deployed was not constant for each SIA round. Incorporating the number of deployed supervisors for each SIA round would have provided another dimension to standardize the average ODK report submissions per round.

## Conclusion

Digital health technologies in public health are now more important than ever in improving the use of data for action and decision-making. The adoption of ODK mobile technology for supportive supervision reporting during SIAs in Nigeria since March 2017 substantially improved reporting rate and supervision coverage, consequentially improving the technical and knowledge support provided to vaccination teams that administered poliovirus vaccines to children under 5 years of age from house-to-house in Nigeria. The mobile technology addressed the drawbacks of paper-based checklists. It enabled a more user-friendly data-capturing process for supervisors of vaccination teams in the field, real-time reporting, timely data analysis on campaign implementation quality and dissemination of feedback each campaign day, improved accountability and geo-location evidence of places visited for supervision with GPS coordinates data capture, spatial data triangulation with other geospatial SIA datasets, and better data management of supportive supervision data. All the benefits experienced with the adoption of ODK mobile digital technology could not be attained or were very limited with paper-based reporting.

We recommend that public health programs consider deploying a mobile data collection technology approach over the use of paper-based forms for large-scale data collection activities, referencing the lessons from the Nigerian polio program in this study. There are other mobile data collection technologies and applications beyond ODK that exist. Adopting any of such technologies will require consideration of unique local conditions and the context of public health activities. However, similar operational and programmatic improvements as documented in this paper will probably be achieved for any mobile data collection technology considered.

**Disclaimer:** the findings and conclusions in this report are those of the authors and do not necessarily represent the official position of the U.S. Centers for Disease Control and Prevention.

### What is known about this topic

- Supportive supervision is a valuable programmatic strategy used to impart knowledge and help improve the skills of health workers in implementing their tasks and advance health outcomes in African countries;
- Open Data Kit (ODK) is an open-source mobile technology application that has gained popularity in recent years and is becoming widely used to capture and manage data in various public health programs or projects, especially in low-resource settings or low-to-middle-income countries.

### What this study adds

- Detailed comparative advantages of using ODK technology over paper forms to report supportive supervision conducted during polio Supplementary Immunization Activities (SIA) in Nigeria;
- The impact of adopting ODK to strengthen supervision reporting, coverage, and consequentially quality of supervision to guide vaccination teams in the service-delivery of oral poliovirus vaccine during polio SIAs in Nigeria.
